# Development of Marker-Free Insect-Resistant Indica Rice by *Agrobacterium tumefaciens*-Mediated Co-transformation

**DOI:** 10.3389/fpls.2016.01608

**Published:** 2016-10-27

**Authors:** Fei Ling, Fei Zhou, Hao Chen, Yongjun Lin

**Affiliations:** National Key Laboratory of Crop Genetic Improvement, National Center of Plant Gene Research, Huazhong Agricultural UniversityWuhan, China

**Keywords:** co-transformation, *cry2A*, insect-resistant, marker-free, transgenic rice

## Abstract

*Agrobacterium*-mediated co-transformation is an efficient strategy to generate marker-free transgenic plants. In this study, the vectors pMF-2A^∗^ containing a synthetic *cry2A^∗^* gene driven by maize ubiquitin promoter and pCAMBIA1301 harboring hygromycin phosphotransferase gene (*hpt*) were introduced into Minghui86 (*Oryza sativa* L. ssp. *indica*), an elite *indica* restorer line. Two independent transformants containing both the *cry2A^∗^* gene and *hpt* gene were regenerated. Several homozygous marker-free transgenic progenies were derived from family 2AH2, and three of them were selected for further insect bioassay in the laboratory and field. Insect-resistance assays revealed that all the three transgenic lines were highly resistant to striped stem borer (*Chilo suppressalis*), yellow stem borer (*Tryporyza incertulas*) and rice leaf folder (*Cnaphalocrocis medinalis*). The measurement of Cry2A protein concentration showed that Cry2A protein was stably expressed in leaves and stems of homozygous transgenic lines and their hybrids. The yields of the marker-free homozygous transgenic lines and their hybrids were not significantly different from those of their corresponding controls. Furthermore, the results of flanking sequence isolation showed that the T-DNA in line 8-30 was integrated into the intergenic region of chromosome 2 (between *Os02g43680* and *Os02g43690*). These results indicate that the marker-free transgenic rice has the potential for commercial production.

## Introduction

Rice is one of the most important staple food crops in the world. Insect damage can cause severe rice yield loss up to 10% every year (Chen et al., 2009). Rice leaf folder, striped stem borer (SSB), and yellow stem borer (YSB) are three major pests in rice production. Traditionally, chemical pesticides are used to control these pests, which cause a series of problems, such as environmental pollution, pesticide residue and increase of production cost. Bt proteins have been widely used as a bioinsecticide and the *Bt* genes have been introduced into rice, corn, cotton, soybean, and other crops to control pests, such as Lepidoptera pests. Transgenic crops such as corn and cotton that express Bt proteins for commercial application have brought great economic benefits (Clive, 2012).

Currently, *Agrobacterium tumefaciens*-mediated genetic transformation is widely applied to the development of transgenic crops, and antibiotic or herbicide-resistant selectable marker genes (SMGs) are used to select transformed cells in the transformation process. SMGs are redundant after transformation, and are not conducive to continuous transformation and safety. Thus, a series of marker-free strategies were developed, such as biolistic method (Tu et al., 2000; Shiva Prakash et al., 2009) and *A. tumefaciens*-mediated co-transformation, site-specific recombination (Li et al., 2007; Qiu et al., 2010; Sengupta et al., 2010; Yu et al., 2013; Woo et al., 2015b), multi-auto-transformation vector system (Endo et al., 2002; Khan et al., 2006; Scaramelli et al., 2009), transposon-mediated method (Gao et al., 2015), intrachromosomal recombination (Zubko et al., 2000), and PCR-based method (de Vetten et al., 2003; Woo et al., 2015a). There are three approaches for the co-transformation: one plasmid harboring two T-DNAs in one *Agrobacterium* strain (Breitler et al., 2004; Matheka et al., 2013); two T-DNAs located on two plasmids in the same *Agrobacterium* strain (Parkhi et al., 2005; Sripriya et al., 2008); two T-DNAs in separate *Agrobacterium* strains (Dutt et al., 2012). The approach of ‘two T-DNAs in separate *Agrobacterium* strains’ was adopted in this study, because the marker-free progeny has the target T-DNA sequence but does not include other redundant sequences.

Similar to the application of chemical pesticides, wide cultivation of Bt crops also can cause the insects to evolve resistance to *Bt* genes. The *cry1A* genes such as *cry1Aa, cry1Ab*, and *cry1Ac* are used to control Lepidoptera pest in transgenic rice at present (Nayak et al., 1997; Cheng et al., 1998; Ye et al., 2001; Khanna and Raina, 2002). It has been reported that Cry1Aa, Cry1Ab, and Cry1Ac have cross-resistance because of the common binding sites (Escriche et al., 1997). Thus, these proteins cannot be applied to develop *Bt* gene-stacking rice. Studies focused on transgenic insect-resistant rice with other types of *Bt* genes are rather limited so far, and there have been only a few studies about marker-free transgenic insect-resistant rice (Tu et al., 2000; Kumar et al., 2010). Several previous studies have shown that the Cry2A and Cry1A proteins have different binding sites in brush border membrane vesicles (BBMVs) and exhibit no cross-resistance (Lee et al., 1997; Karim and Dean, 2000; Alcantara et al., 2004; Gouffon et al., 2011). Therefore, *cry2A* gene can be applied to develop marker-free *Bt* transgenic rice. Then, the marker-free *cry2A* transgenic rice can be used as a resistance source for developing gene-pyramiding marker-free transgenic rice, which can delay the evolution of resistance of insects to Bt rice. In this study, the *cry2A^∗^* gene, which was synthesized by rice codon optimization with a 69.45% sequence homology with the original *cry2Aa* gene (Chen et al., 2005), was introduced into an elite *indica* restorer line Minghui86 (MH86) with the purpose of developing marker-free *cry2A* transgenic rice.

## Materials and Methods

### Constructs and Genetic Transformation

pCAMBIA1300 (provided by the Centre for the Application of Molecular Biology in International Agriculture, Australia) was digested by *Bst*XI and *Xho*I to remove the CaMV35s promoter and hygromycin phosphotransferase (*hpt*) gene, and then the *cry2A^∗^* gene driven by ubiquitin promoter (Christensen et al., 1992) was cloned into the multiple cloning sites to obtain the transformation vector pMF-2A^∗^. The T-DNA region is displayed in Supplementary Figure S1. The pMF-2A^∗^ and pCAMBIA1301 were introduced into *A. tumefaciens EHA105*, respectively. The two strains were used to infect an elite *indica* restorer line MH86 together at the ratio of 3:1. The callus culture and genetic transformation procedures were based on the method described by Lin and Zhang (Lin and Zhang, 2005).

### Detection of PCR and Southern Blotting

The *cry2A^∗^* and *hpt* genes were detected by PCR and Southern blotting. The primers for the amplification of *cry2A^∗^* were Cry2A-F (5′-CGTGTCAATGCTGACCTGAT-3′) and Cry2A-R (5′-GATGCCGGACAGGATGTAGT-3′). The primers for the amplification of *hpt* were hpt-F (5′-AGAATCTCGTGCTTTCAGCTTCGA-3′) and hpt-R (5′-TCAAGACCAATGCGGAGCATATAC-3′). The PCR mixture contained 25 ng of template DNA, 2 μl of 10× buffer, 1.5 μl of 2 mM dNTP, 0.2 μl of each primer (10 μM), 0.2 μl of rtaq. The PCR reaction for the amplification of *cry2A^∗^* was implemented at 94°C for 5 min; 32 cycles of 94°C for 30 s, 57°C for 30 s, 72°C for 30 s; 72°C for 7 min. The PCR reaction to amplify *hpt* was implemented at 94°C for 5 min; 32 cycles of 94°C for 30 s, 58°C for 30 s, 72°C for 30 s; 72°C for 7 min.

The total genomic DNA of the transformants was isolated by CTAB method for Southern blotting (Murray and Thompson, 1980). There are *Hin*dIII sites in the T-DNAs of pMF2A^∗^ (Supplementary Figure S1) and pCAMBIA1301, and there is no *Hin*dIII site between the probe (*cry2A^∗^* or *hpt*) and LB of the T-DNA. Therefore, the DNA samples (8 μg) were digested by *Hin*dIII. The plasmids pMF-2A^∗^ and pCAMBIA1301 were also digested by *Hin*dIII and 0.5 ng of digested plasmids was loaded as positive control, respectively. The digested DNA samples were separated by 1% TAE agarose gel and transferred to nylon membrane. Each sample was hybridized by *cry2A^∗^* probe (a PCR-amplified fragment of *cry2A^∗^* by Cry2A-F and Cry2A-R) and *hpt* probe (a PCR-amplified fragment of *hpt* by hpt-F and hpt-R). The hybridization procedures were carried out as described by Southern (Southern, 1975).

### Selection of Marker-Free Homozygous Cry2A^∗^ Lines with Single Copy

The 160 T_1_ generation transgenic plants derived from the T_0_ transgenic plant with single copy of both *cry2A^∗^* and *hpt* were planted. The T_1_ transgenic lines containing *cry2A^∗^* and no *hpt* were selected by PCR analysis, and were harvested individually. Then, the harvested seeds of these T_1_ transgenic lines were sown in seedling bed and *cry2A^∗^* gene was detected in the seedlings (T_2_ generation) through PCR analysis. If the *cry2A^∗^* gene did not segregate in T_2_ generation population derived from the same T_1_ plant, the T_1_ plant was considered as a *cry2A^∗^* homozygous line. Three homozygous lines were used for further assays and the presence of *cry2A^∗^* and absence of *hpt* were confirmed by southern blotting.

### Measurement of Cry2A^∗^ Protein Content

In 2016, the concentrations of Cry2A^∗^ protein in leaves (at tillering, heading and filling stage), stems (at tillering, heading and filling stage) and endosperms (at filling stage) of three transgenic homozygous lines and their hybrids were measured using the enzyme-linked immune sorbent assay (ELISA) kit AP005 from EnviroLogix Inc. (Portland, ME, USA). These hybrids were obtained by crossing the homozygous lines with II-32A, an elite *indica* cytoplasmic male sterility (CMS) line, respectively. Fresh leaf samples of approximately 20 mg were collected and homogenized by grinding in 500 μl of extraction buffer, and fresh stem and endosperm samples of approximately 40 mg were collected and homogenized by grinding in 1 ml of extraction buffer. All of the sample extracts were diluted in appropriate proportions. To quantify the Cry2A protein content, the Cry2Aa Calibrators (1, 5, and 10 ppb) provided by the manufacturer were used to create a standard curve. The Cry2A protein immunoassay procedures followed the protocol of the manufacturer. The optical density values were measured with a microplate reader at 450 nm wavelength. The Cry2A^∗^ protein concentration (ppb) of each diluted sample was obtained from the standard curve. The final content of Cry2A^∗^ protein was calculated according to the following equation: Cry2A^∗^ protein content (μg/g) = [concentration value from the standard curve (ppb) × dilution multiple × extraction buffer volume (ml)]/fresh weight of each sample (mg). To calculate the percentage of Cry2A^∗^ protein, the Bradford assay (Bradford, 1976) was used to quantify the total protein concentration. One-way analysis of variance and the LSD test were used to analyze the significant difference in the mean values between different stages of transgenic lines.

### Insect Assay in the Laboratory

The resistance of the transgenic lines against SSB and YSB was tested in the laboratory. The SSB were fed in our lab, and the eggs of yellow rice borers were collected in the field and hatched in laboratory. The resistance of the three transgenic homozygous lines (MH86 as the susceptible control) and their hybrids (II Youming86 produced by crossing MH86 with II-32A as the susceptible control) was tested. The tested rice stems were collected from field at elongation stage and dissected into 8 cm in length. One glass tube (12 cm in length and 2 cm in diameter), which contained three dissected stems, was set as a biological repeat. Fifteen first-instar larvae of SSB of YSB were placed into the tube and sealed with cotton and black gauze. Three biological repeats were performed for each tested rice line. All of the glass tubes were incubated at 28°C and 80% relative humidity. After 6 days the larvae mortality was investigated. One-way analysis of variance and the LSD test were used to compare the significant difference in the mean values between transgenic lines and corresponding controls.

### Insect Assay in the Field

Insect resistance of the three transgenic homozygous lines in the field was evaluated at Huazhong Agricultural University experimental field in 2011, Wuhan, China. The transformation receptor MH86 was planted as susceptible control. The seeds of all tested materials were sown on rice seedling bed in June 6, 2011 and the seedlings were transplanted to paddy field in July 2. The seedlings of the four rice materials were distributed according to a randomized block design with three replications. Each replication consisted of 24 plants of each material in two rows, with a planting distance of 20 cm within a row and 26.7 cm between rows. The experimental plots were bordered by three rows of non-transgenic *indica* rice plants as protection. The experimental field was not sprayed with chemical pesticides during the entire growth period.

To evaluate the resistance of transgenic lines against stem borers in the field, each rice plant was infested with about 15 first-instar larvae of YSB at the booting stage. Rice dead hearts caused by stem borers was investigated after two weeks of artificial infestation. Meanwhile, leaf folds caused by natural infestation of rice leaf folder were counted. The insect resistance of transgenic hybrids and II Youming86 was evaluated in the field in 2013 using the similar method in 2011 except for that the planting distance was 17 cm within a row and 26.7 cm between rows. Because the eggs of YSB were not collected in this year, the insecticidal activity of the hybrids was evaluated under natural infestation in the experimental paddy. Non-repeated two-factor analysis of variance and LSD test were used to compare the significant difference in the mean values between transgenic lines and corresponding controls.

### Evaluation of Agronomic Performance in the Field

The design of experiments for evaluating the agronomic performance of the transgenic homozygous lines and their hybrids were conducted as described above in the insect assay in the field in 2011 and 2013, respectively. Chemical insecticides were sprayed during the growth period to protect the crop. The plant height of 20 plants in the middle of each plot was measured in the field at maturity, and these plants were harvested to record the number of panicles per plant, panicle length, number of grains per panicle, 1000-grain weight, seed setting rate and yield per plant. Non-repeated two-factor analysis of variance and LSD test were also used to compare the significant difference in the mean values between transgenic lines and corresponding controls.

### Isolation of Flanking Sequences

Thermal asymmetric interlaced polymerase chain reaction (TAIL PCR) was adopted for isolating the flanking sequences of the T-DNA integration site in the transgenic plant. Three nested specific primers (SP1, SP2, and SP3) near the T-DNA right border (RB) and degenerate primer (AD8) were designed (Supplementary Table S1). The PCR mixture and reaction programs were based on the method described by Liu and Chen (2007). The tertiary amplification product was cloned to pEasy-T3 vector and sequenced. The flanking sequence of T-DNA RB flanking region was obtained by analysis of the sequencing results, and then the sequence was used for blastn in the bioinformatic website^1^ in order to determine the T-DNA integration site in rice genome. Meanwhile, the putative flanking sequence of T-DNA left border (LB) flanking region was obtained through querying in bioinformatic website ^2^ according to the blastn results. The genome primer 2AH2-tF based on the putative flanking sequence of LB and primer Tail-L1 in vector pMF-2A^∗^ were designed (Supplementary Table S1), and the PCR product of the two primers was sequenced to confirm the flanking sequence of LB.

## Results

### Genetic Transformation and DNA Assay

**FIGURE 1:**
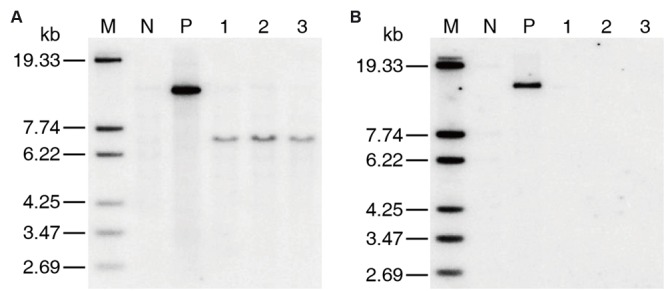
**Southern blotting of T_2_ generation marker-free transgenic homozygous lines. (A)** *cry2A^∗^* probe, **(B)** *hpt* probe. M DNA Marker, N MH86 control, P positive plasmid control, 1 line 7-61, 2 line 8-30, 3 line 8-62.

Only two positive co-transformation plants, which were named as 2AH1 and 2AH2, respectively, were obtained in T_0_ generation by co-transformation of MH86 callus. The copy number of transgenic plants was confirmed by southern blotting. The results showed that both *cry2A^∗^* and *hpt* genes were single-copy in the 2AH2 family (Supplementary Figure S2). Three marker-free homozygous lines containing *cry2A^∗^* (named as 7-61, 8-30, and 8-62) derived from 2AH2 were selected by PCR and southern blot analysis (**Figure 1**).

### Cry2A^∗^ Concentrations of Transgenic Homozygous Lines and Hybrids

**FIGURE 2:**
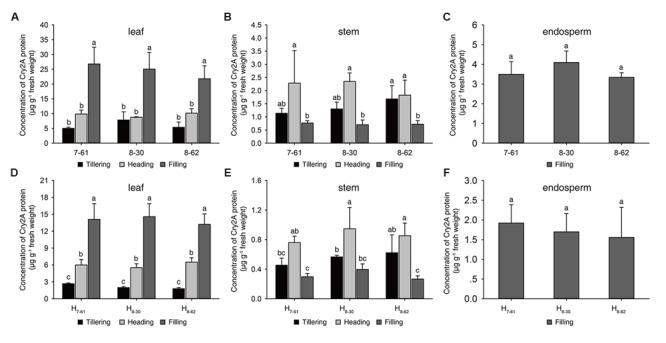
**Cry2A protein concentrations in three homozygous lines and their hybrids. (A-C)** homozygous lines, **(D-F)** hybrids. a, b, c: significant difference at *P* < 0.05. Error bars indicate SD (*n* = 3).

In 2016, the Cry2A protein contents and the percentage of Cry2A protein in soluble protein of transgenic homozygous lines were measured at tillering, heading and filling stage (**Figures 2A**-C; Supplementary Table S2). In leaves, the concentrations of Cry2A protein ranged from 5.01 μg g^-1^ to 26.79 μg g^-1^ fresh weight, and the percentage of Cry2A protein ranged from 0.016 to 0.099% in soluble protein. In stems, the Cry2A protein concentrations ranged from 0.70 μg g^-1^ to 2.35 μg g^-1^ fresh weight, and the percentage of Cry2A protein ranged from 0.018 to 0.039% in soluble protein. In endosperms, the contents of Cry2A protein ranged from 3.34 μg g^-1^ to 4.09 μg g^-1^ fresh weight, and the percentage of Cry2A protein ranged from 0.026 to 0.031% in soluble protein. These results indicated that the Cry2A protein was stably expressed in the three transgenic homozygous lines at each growth stage.

To evaluate the commercial potential of the three transgenic homozygous lines, three hybrids, namely H_7-61_, H_8-30_, and H_8-62_, were produced from the crossing of II-32A with the transgenic homozygous lines, respectively. The Cry2A protein content and its proportion in soluble protein in the Bt hybrids were also measured in 2016. In the hybrids, the concentrations of Cry2A protein in leaves ranged from 1.79 μg g^-1^ to 14.56 μg g^-1^ fresh weight, and the proportion of Cry2A protein ranged from 0.007 to 0.049% in soluble protein. In stems, the Cry2A protein concentrations ranged from 0.27 μg g^-1^ to 0.95 μg g^-1^ fresh weight, and the proportion of Cry2A protein ranged from 0.008 to 0.017% in soluble protein. In endosperms, the contents of Cry2A protein ranged from 1.56 μg g^-1^ to 1.92 μg g^-1^ fresh weight, and the proportion of Cry2A protein ranged from 0.012 to 0.013% in soluble protein (**Figures 2D-F** and Supplementary Table S3). These results suggested that the Cry2A contents in the hybrids were lower than in the homozygous lines.

### Insect-Resistance of Transgenic Rice in the Laboratory

**FIGURE 3:**
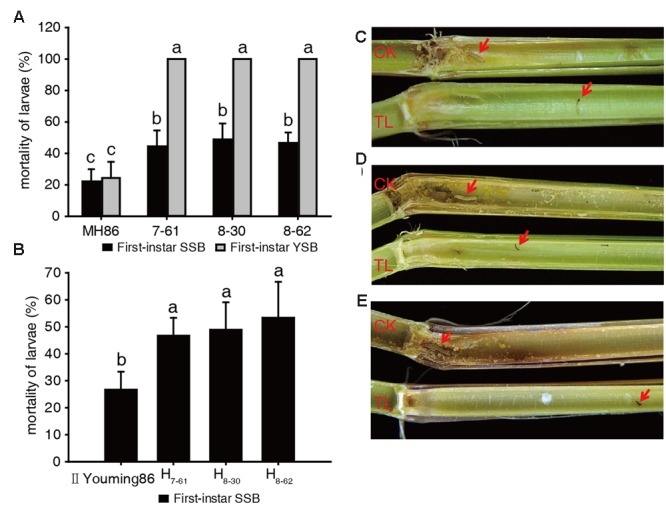
**Insect-resistance assay of transgenic materials in the laboratory.** Larvae mortality of SSB and YSB fed on three transgenic homozygous lines **(A)**; larvae mortality of SSB fed on three transgenic hybrids **(B)**; resistance of transgenic homozygous lines against first-instar SSB **(C)** and YSB **(D)**; resistance of transgenic hybrids against first-instar SSB **(E)**. a, b, c: significant difference at *P* < 0.05. Error bars indicate SD (*n* = 3).

In the laboratory assay of the insecticidal activity of the transgenic homozygous lines, the larvae mortality of SSB or YSB fed on non-transgenic control MH86 stems was approximately 22% after 6 days, and the surviving larvae showed normal development. In addition, the MH86 stems were severely damaged and plenty of frass was observed. The larvae mortality of SSB fed on transgenic rice stems was about 50%, and the surviving larvae showed developmental retardation: their body length was significantly shorter than that of survival from non-transgenic control MH86. Besides, there was a smaller amount of frass (**Figures 3A,C**). All YSB larvae fed on transgenic rice stem were killed after 6 days, and the stems suffered from slight damage and little frass (**Figures 3A,D**). These results revealed the high resistance of transgenic homozygous lines against YSB.

In the insect-resistance assay of the hybrids, the stem cuttings of II Youming86 showed similar level of damage as the stems of MH86, and the mortality of SSB larvae was about 25%. Besides, the surviving larvae developed normally. However, the three transgenic hybrids could kill about 50% of the SSB larvae, and the development of the surviving larvae was retarded (**Figures 3B,E**). The results indicated that the Cry2A protein content of the transgenic hybrids was lower than that of transgenic homozygous lines, but the hybrid stems showed equivalent insecticidal activity with their parents.

### Insect-Resistance of Transgenic Rice in the Field

**FIGURE 4:**
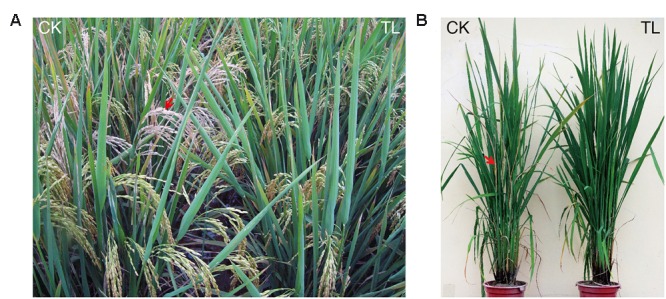
**Insect resistance performance of transgenic rice in the field. (A)** Resistance of transgenic homozygous lines against rice leaf folder and rice stem borer. CK: MH86, TL: transgenic line. Red arrow indicates white head. **(B)** Resistance of transgenic hybrids against rice stem borer. CK: II Youming86, TL: transgenic hybrid. Red arrow indicates dead hearts.

In the insect-resistance assay of the transgenic homozygous lines in the field in 2011, the leaves of non-transgenic control MH86 showed serious folds caused by rice leaf folders. Averagely two leaves were recorded as being folded per tiller and the percentage of tillers suffering from damage per plant was up to 99%. However, the percentage of tillers damaged in the three transgenic homozygous lines ranged from 4.78 to 8.25% and the leaves were only slightly damaged (0.06-0.11 leaves per tiller). Very few white heads were observed in the transgenic homozygous lines in contrast to the white heads in non-transgenic control MH86 per plant (11.84%) (**Table 1**; **Figure 4A**).

**Table 1:** Resistance of transgenic homozygous lines against rice leaf folder and stem borers in the field under artificial infestation combined with natural infestation in 2011.

Lines	Number of folded leaves per tiller	Percentage of tillers damaged by leaffolders (%)	Percentage of white heads (%)
MH86	2.04 ± 0.10	99.02 ± 0.55	11.84 ± 3.34
7-61	0.11 ± 0.02^∗∗^	8.23 ± 0.65^∗∗^	0.00 ± 0.00^∗∗^
8-30	0.11 ± 0.01^∗∗^	8.25 ± 1.16^∗∗^	0.19 ± 0.34^∗∗^
8-62	0.06 ± 0.04^∗∗^	4.78 ± 2.59^∗∗^	0.00 ± 0.00^∗∗^

*Values are the means ± SD (*n* = 3). ^∗∗^Significantly different from MH86 (*P* < 0.01).*

The resistance of the transgenic hybrids against target insects in the field was evaluated under natural infestation in 2013. The damage caused by rice leaf folders was not severe in this year. 15.46% of plant tillers suffered from damage in susceptible control II Youming86, and the transgenic hybrids showed only slight damage (**Table 2**). Some dead hearts caused by rice stem borers were observed in II Youming86 (**Figure 4B**), while almost no damage was observed in the transgenic hybrids. These results revealed that the transgenic rice was highly resistant to rice leaf folder and rice stem borer in the field.

**Table 2:** Resistance performance of transgenic hybrids in the field after natural infestation in 2013. Values are the means ± SD (*n* = 3).

Lines	Number of folded leaves per tiller	Percentage of tillers damaged by leaffolders (%)
II Youming86	0.18 ± 0.06	15.46 ± 4.60
H_7-61_	0.02 ± 0.00^∗∗^	1.56 ± 0.42^∗∗^
H_8-30_	0.02 ± 0.00^∗∗^	1.74 ± 0.35^∗∗^
H_8-62_	0.01 ± 0.01^∗∗^	1.01 ± 0.68^∗∗^

*^∗∗^Significantly different from II Youming86 (*P* < 0.01).*

### Agronomic Performance of Transgenic Rice in the Field

Some agronomic traits of the three transgenic homozygous lines showed different degrees of variation. The panicle length and 1000-grain weight were significantly lower than those of non-transgenic control MH86 (*P* < 0.01), but the numbers of grains per panicle of line 7-61 and line 8-30 were significantly larger than that of MH86 (*P* < 0.05). All the transgenic homozygous lines showed no change in yield compared with wide-type MH86 (**Table 3**). The three transgenic hybrids showed little difference with non-transgenic control II Youming86 in agronomic performance, especially the hybrid of line 8-30 (**Table 4**).

**Table 3:** Agronomic performance of transgenic homozygous lines in the field.

Lines	Plant height (cm)	Panicle length (cm)	Panicles per plant	Grains per panicle	Seed setting rate (%)	1000-grain weight (g)	Yield per plant (g)
MH86	131.6 ± 3.0	28.6 ± 0.3	12.2 ± 1.1	169.7 ± 4.8	87.3 ± 2.7	29.7 ± 0.5	62.2 ± 1.9
7-61	128.2 ± 3.4	26.7 ± 0.4^∗∗^	12.8 ± 0.4	179.8 ± 2.1^∗^	87.5 ± 1.5	27.1 ± 0.5^∗∗^	62.1 ± 2.1
8-30	130.1 ± 1.3	27.2 ± 0.5^∗∗^	13.4 ± 0.5^∗^	177.2 ± 0.9^∗^	86.7 ± 2.3	27.0 ± 0.4^∗∗^	64.4 ± 3.2
8-62	125.9 ± 1.1^∗^	26.2 ± 0.3^∗∗^	13.4 ± 0.1^∗^	171.2 ± 5.6	90.1 ± 1.0	25.8 ± 0.4^∗∗^	58.8 ± 2.8
LSD_0.05_	3.9	0.8	1.1	7.0	4.0	0.9	4.1
LSD_0.01_	6.0	1.3	1.7	10.6	6.0	1.3	6.1

*Values are the means ± SD (*n* = 3). ^∗^Significantly different from MH86 (*P* < 0.05); ^∗∗^Significantly different from MH86 (*P* < 0.01).*

**Table 4:** Agronomic performance of transgenic hybrids in the field.

Lines	Plant height (cm)	Panicle length (cm)	Panicles per plant	Grains per panicle	Seed setting rate (%)	1000-grain weight (g)	Yield per plant (g)
II Youming86	118.8 ± 3.2	28.2 ± 0.4	11.3 ± 0.6	163.8 ± 4.2	80.4 ± 0.6	26.1 ± 0.2	47.5 ± 3.3
H_7-61_	117.8 ± 2.3	26.9 ± 0.2^∗^	11.9 ± 1.1	152.8 ± 5.4^∗^	79.5 ± 4.3	25.6 ± 0.4	46.0 ± 2.6
H_8-30_	119.3 ± 0.2	27.5 ± 1.3	12.0 ± 1.0	158.0 ± 7.4	79.5 ± 1.3	25.8 ± 0.4	48.9 ± 3.4
H_8-62_	115.6 ± 1.5^∗^	27.6 ± 1.1	11.8 ± 0.2	154.6 ± 2.2	79.8 ± 1.1	25.5 ± 0.1	46.4 ± 1.6
LSD_0.05_	2.8	1.3	1.3	9.8	3.2	0.7	4.5
LSD_0.01_	4.0	1.8	1.8	13.7	4.4	1.0	6.3

*Values are the means ± SD (*n* = 3). ^∗^Significantly different from II Youming 86 (*P* < 0.05).*

### Flanking Sequences of T-DNA Integration Site in Rice Genome

**FIGURE 5:**
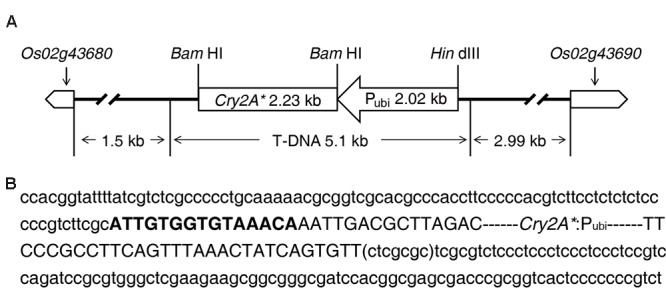
**Information of T-DNA integration site in transgenic line 8-30. (A)** T-DNA inserted into rice chromosome 2 between gene *Os02g43680* and gene *Os02g43690*. **(B)** Nucleotide sequences of T-DNA integration site. The two flanking sequences of T-DNA are displayed in lowercase letter. The sequence in bracket is the deletion sequence of genome. The T-DNA sequence is shown in capital letter. Bold capital letters represent the truncated T-DNA left border (LB). T-DNA right border (RB) is missing in line 8-30.

The sequence analysis results of TAIL-PCR and subsequent routine PCR revealed that one complete T-DNA copy containing cry2A* gene was inserted into rice genome chromosome 2 in line 8-30. The information of the genes nearby T-DNA integration site was obtained by querying the flanking sequence in the bioinformatics website^3^. The results indicated that the T-DNA LB and transcription initiation site of gene *Os02g43680* had a distance of about 1.5 kb, and the T-DNA RB was 2.99 kb away from the transcription initiation site of gene *Os02g43690* (**Figure 5A**); besides, the T-DNA insertion led to a 7-bp deletion of rice genome (ctcgcgc) (**Figure 5B**).

## Discussion

In this study, a synthetic cry2A* gene was introduced into an elite *indica* restorer MH86 via *Agrobacterium*-mediated co-transformation. Subsequently, three single-copy marker-free transgenic homozygous lines were selected by PCR analysis and Southern blotting. The three transgenic lines not only exhibited high resistance against rice leaf folder and rice stem borer (**Figures 3** and **4**), but also showed no significant difference in yield compared with their corresponding controls under the spraying of chemical insecticides during the entire growth period (**Tables 3** and **4**). In the laboratory insect assay, the larvae mortality of SSB was lower than that of YSB. This result was consistent with the previous study in our laboratory (Yang et al., 2011), which indicated that SSB is less susceptible to Cry2A protein than YSB. The difference in susceptibility is mainly due to the difference in Cry2A protein binding sites and binding affinities in the BBMVs of the insect midguts (Lee et al., 1997; Alcantara et al., 2004). It has been reported that the LC_50_ of Cry2A protein in the artificial diet for SSB was significant higher than that for YSB (Lee et al., 1997), which also suggests that Cry2A protein is more toxic to YSB than to SSB.

As we know, the presence of SMGs in transgenic plants can cause a variety of problems. Herbicide resistance genes, which can endow the transgenic plants with herbicide tolerance, are often used as selectable markers in transformation. However, the majority of SMGs are useless after the generation of transgenic plants, and their presence will increase the metabolism burden. More importantly, the presence of SMGs in transgenic plants also brings about safety concerns, particularly in rice, which is directly consumed by humans. In the past two decades, a number of marker-free plant transformation strategies have been reported and commonly used, including the *Agrobacterium*-mediated co-transformation and site-specific recombination. In this study, the approach ‘two T-DNAs in separate *Agrobacterium* strains’ was adopted. Two *Agrobacterium* strains, respectively, harboring the vector with SMG (*hpt* gene) and the vector with insect-resistant gene cry2A* were used for genetic transformation, and marker-free transgenic rice was developed successfully, which might address the consumers’ concerns about food safety of transgenic rice.

With the large-scale cultivation of Bt transgenic crops, the insect populations that evolve resistance to Bt proteins have been found in the field. The evolution of insect resistance is mainly due to the mutation of cadherin, which binds with Bt protein in the midgut of insect. Gene pyramiding is an effective strategy to deal with insect resistance. The *cry1A* genes, including *cry1Ab, cry1Ac*, and the fusion gene *cry1Ab/Ac*, are most commonly used in transgenic rice so far, especially in marker-free transgenic rice. The proteins encoded by *cry1A* genes have common binding sites in the BBMVs of insect midgut, and they have cross-resistance between each other. Therefore, other types of *Bt* genes should be combined with *cry1A* genes to develop gene-stacking Bt transgenic rice. In this study, the marker-free transgenic rice expressing Cry2A protein, which showed different binding sites with Cry1A protein in the midgut of SSB or YSB, can be used to cross with marker-free Cry1A transgenic rice for developing marker-free transgenic rice with double *Bt* genes to delay the evolution of insect resistance. The marker-free *cry1Ab/Ac* transgenic *indica* rice TT51 developed previously by our laboratory has been issued with security certificate again by Chinese government in 2014, indicating the great commercialization potential of marker-free Bt transgenic rice. Thus, it can be speculated that the marker-free cry2A* transgenic rice developed in this study has the similar potential for commercialization.

The results of flanking sequence isolation revealed that T-DNA containing cry2A* was integrated into the intergenic region of chromosome 2 (**Figure 5**). Although the transgenic homozygous lines were significantly different from non-transgenic wide-type MH86 in several agronomic traits (**Table 3**), the transgenic hybrids showed little difference from the non-transgenic control II Youming86 in agronomic performance (**Table 4**). These results suggest that the variations of agronomic traits in transgenic homozygous lines might be caused by the somatic mutation in the tissue culture. However, most of these variations were recovered in the hybrids. Yang et al. (2011) reported the similar results when they recorded the agronomic traits of the hybrids derived from four single *Bt*-gene transgenic rice lines and 10 two *Bt*-gene transgenic rice lines crossing with an *indica* CMS Zhenshan97A, respectively. These results suggest that the consecutive backcross of the transgenic homozygous line 8-30 with the wide-type MH86 can eliminate the somatic variations (Bregitzer et al., 2008; Yang et al., 2012).

## Author Contributions

FL, FZ, HC, and YL designed the experiments. FL performed the experiments, collected, and analyzed the data. FL and YL wrote the manuscript.

## Conflict of Interest Statement

The authors declare that the research was conducted in the absence of any commercial or financial relationships that could be construed as a potential conflict of interest.

## Footnotes

1 http://rice.plantbiology.msu.edu/

2 http://rice.plantbiology.msu.edu/cgi-bin/gbrowse/rice/#search

3 http://rice.plantbiology.msu.edu/cgi-bin/gbrowse/rice/\#search
