# Supplementary material for: Development of Marker-Free Insect-Resistant Indica Rice by Agrobacterium tumefaciens-Mediated Co-transformation
Source: Front Plant Sci. 2016 Oct 27;7:1608. doi: 10.3389/fpls.2016.01608 (PMC5081342; doi:10.3389/fpls.2016.01608)
Supplement: Supplementary file 1 [file Table_1.DOCX]

**Table S1. Primers used in Tail-PCR.**

| Primer name | DNA sequence (5’—3’) |
| --- | --- |
| SP1 | CAACTTAATCGCCTTGCAGCAC |
| SP2 | CTGGCGTAATAGCGAAGAGGC |
| SP3 | CAACAGTTGCGCAGCCTGAAT |
| AD8 | AGWGNAGWANCAWAGG |
| 2AH2-tF | TCATCCGGAGTCACAAGGTCT |
| Tail-L1 | CTATAGGGTTTCGCTCATGTGTTG |

**Table S2. Percentage of Cry2A protein in soluble protein in three homozygous lines.**

| Lines | Leaves | | |  | Stems | | |  | Endosperms |
| --- | --- | --- | --- | --- | --- | --- | --- | --- | --- |
|  | Tillering (%) | Heading (%) | Filling  (%) |  | Tillering (%) | Heading (%) | Filling  (%) |  | Filling  (%) |
| 7-61 | 0.020±0.003 | 0.022±0.000 | 0.099±0.019 |  | 0.020±0.005 | 0.022±0.011 | 0.034±0.002 |  | 0.031±0.008 |
| 8-30 | 0.026±0.008 | 0.016±0.001 | 0.082±0.035 |  | 0.021±0.002 | 0.018±0.002 | 0.028±0.005 |  | 0.026±0.002 |
| 8-62 | 0.019±0.003 | 0.021±0.005 | 0.062±0.017 |  | 0.039±0.020 | 0.018±0.006 | 0.025±0.005 |  | 0.027±0.003 |

**Table S3.** **Proportion of Cry2A protein in soluble protein in three hybrids.**

| Lines | Leaves | | |  | Stems | | |  | Endosperms |
| --- | --- | --- | --- | --- | --- | --- | --- | --- | --- |
|  | Tillering (%) | Heading (%) | Filling  (%) |  | Tillering (%) | Heading (%) | Filling  (%) |  | Filling  (%) |
| H_7-61_ | 0.009±0.001 | 0.010±0.001 | 0.049±0.001 |  | 0.011±0.001 | 0.008±0.001 | 0.014±0.002 |  | 0.013±0.004 |
| H_8-30_ | 0.007±0.001 | 0.010±0.002 | 0.042±0.007 |  | 0.012±0.003 | 0.008±0.002 | 0.017±0.003 |  | 0.012±0.003 |
| H_8-62_ | 0.007±0.001 | 0.012±0.001 | 0.043±0.008 |  | 0.012±0.003 | 0.008±0.001 | 0.011±0.002 |  | 0.012±0.005 |
