# Supplementary material for: Development of Marker-Free Insect-Resistant Indica Rice by Agrobacterium tumefaciens-Mediated Co-transformation
Source: Front Plant Sci. 2016 Oct 27;7:1608. doi: 10.3389/fpls.2016.01608 (PMC5081342; doi:10.3389/fpls.2016.01608)
Supplement: Supplementary file 2 [file Image_1.PDF]

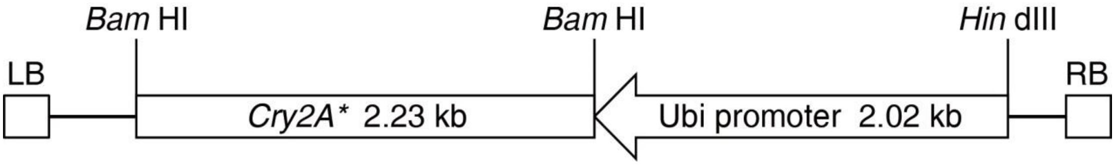

Figure S1. T-DNA region of transformation vector pMF-2A*.

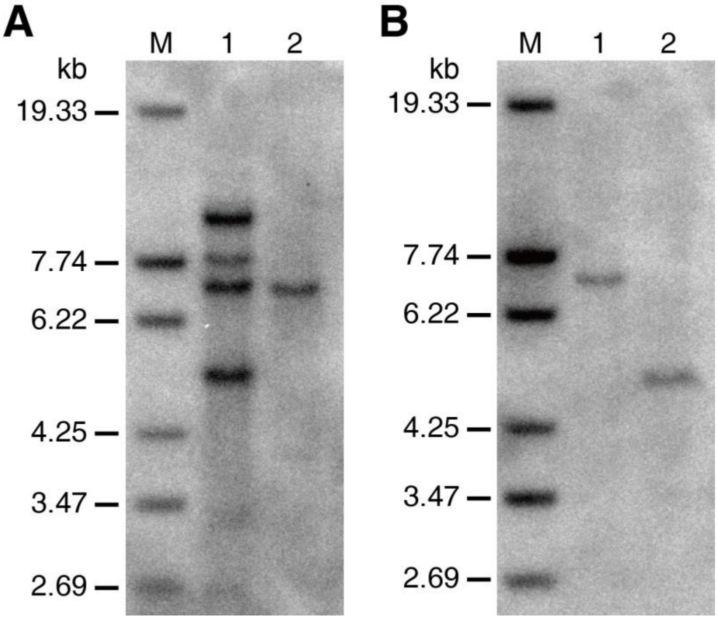

Figure S2. Southern blotting of T_0 generation transgenic plants. (**A**) *cry2A** probe; (**B**) *hpt* probe. M, DNA Marker; 1, family 2AH1; 2, family 2AH2.
